# Microstructure and Optical Properties of Y_1.8_La_0.2_O_3_ Transparent Ceramics Prepared by Spark Plasma Sintering

**DOI:** 10.3390/ma18071389

**Published:** 2025-03-21

**Authors:** Junming Luo, Xu Huang, Liping Deng

**Affiliations:** School of Materials Science and Engineering, Nanchang Hangkong University, Nanchang 330063, China; 17850858905@163.com (X.H.);

**Keywords:** Y_2_O_3_ transparent ceramics, spark plasma sintering, LiF, optical transmittance

## Abstract

Yttrium oxide ceramic is an excellent optical material widely used in lasers, scintillators, and upconversion luminescence. In this study, LiF was employed as an additive to generate volatile gases (CF)_n_ to effectively inhibit carbon contamination and act as a sintering aid to accelerate densification during the spark plasma sintering (SPS) process. The effects of sintering temperature and annealing time on the transmittance of Y_1.8_La_0.2_O_3_ transparent ceramics were systematically investigated. Results indicate that excessive LiF addition reduces the transmittance of Y_1.8_La_0.2_O_3_ transparent ceramics due to the precipitation of F^−^ ions at grain boundaries, forming a secondary phase. For the Y_1.8_La_0.2_O_3_ ceramics with 0.3 wt.% LiF, transmittance initially increases and then decreases with rising sintering temperature, reaching a maximum value of 78.10% in the UV region at 1550 °C. Under these conditions, the average particle size and relative density are 10–30 μm and 99.36%, respectively. Oxygen vacancies within the ceramics act as defects that degrade transmittance. Proper annealing in air reduces oxygen vacancy content, thereby improving transmittance. After annealing at 900 °C for 3 h, the maximum transmittance of Y_1.8_La_0.2_O_3_ ceramics with 0.3 wt.% LiF increases to 82.67% in the UV region, accompanied by a 5.68% reduction in oxygen vacancy concentration.

## 1. Introduction

Y_2_O_3_ ceramic is an excellent optical material with a high melting point, good chemical stability, high thermal conductivity [1], and a wide forbidden band. With high optical transmittance and low phonon energy in the 0.23–8.0 μm band range [2], it is widely used for applications in scintillators [3], upconversion luminescence [4], and other materials.

The sintering-resistant properties of Y_2_O_3_ ceramics [5] result in significant challenges in fabricating dense samples with optimal optical quality through conventional preparation methods. Insufficient densification leads to light scattering phenomena, which are primarily governed by two distinct mechanisms: Rayleigh scattering [6] and Mie scattering [7]. The Mie scattering theory, established by German physicist Gustav Mie in 1908, provides a theoretical framework for describing the scattering phenomenon when light interacts with defect structures whose dimensions are comparable to the wavelength of incident radiation [8]. In transparent ceramics, these light-scattering defect structures predominantly comprise residual pores and secondary phases located at grain boundaries. Based on the Mie scattering theory, Apetz et al. [9] find that the transmittance of the alumina transparent ceramics can be improved obviously when the size of the pores is less than one-tenth of the wavelength of the incident light because the light scattering is significantly attenuated. Similar studies based on the Mie scattering theory have been carried out in other transparent ceramic systems, such as ZrO_2_ [10] and Y_2_O_3_–ZrO_2_ [11]. In order to obtain the Y_2_O_3_ ceramic samples with high density at lower temperatures, researchers began to search for some sintering aids that can accelerate the densification of ceramics. So far, many kinds of sintering aids have been found, such as La_2_O_3_ [12], LiF [13], ZrO_2_ [14], etc. However, it has also been found that the promotion of densification is often accompanied by the degradation of mechanical properties. For example, high-density ceramics can be prepared at lower sintering temperatures by adding LiF, but the hardness of the ceramics will decrease due to the rapid growth of grains [15].

Spark Plasma Sintering (SPS), which is known as pulse current sintering, can be used to prepare transparent ceramic [16,17]. Compared to vacuum sintering [18], microwave sintering [19], hot-press sintering [20], and other methods, it provides a method to obtain a full-density ceramic with controllable microstructure at relatively low temperatures and in a short time. Chaim R et al. [21] studied the effect of SPS conditions with nanocrystalline Y_2_O_3_ powders on the densification behavior and microstructure evolution. They found that the competition between densification and grain growth was dominated by grain growth when the sintering temperature exceeded 1400 °C. According to the research of Koji Morita [22], the SPS heating rate also had a significant effect on the transmittance of Y_2_O_3_ transparent ceramics. The transmittance prepared by rapid heating at 100 °C/min is 66% at 700 nm visible wavelength, while it was only 46% by slow heating at 10 °C/min.

Carbon contamination is also very obvious due to the carbon-rich environment, such as graphite mold, graphite foil, etc. Joshua Gild et al. [23] used a Ti foil to replace the graphite foil, which could effectively reduce the carbon contamination of Y_2_O_3_ transparent ceramics. Sakajio M et al. [24] used Mo and Ta foils to replace the graphite foil to protect the diffusion of carbon. After investigating various experimental combinations to reduce carbon contamination, they found that Ta foils were more effective than Mo foils in YAG transparent ceramics with higher transparency. Yuxin Pan et al. [25] prepared Yb,Ho:Y_2_O_3_ transparent ceramics with transmittance greater than 80% in the wavelength of 1.5 μm by adding LiF to reduce carbon pollution.

Y_1.8_La_0.2_O_3_ transparent ceramics have been researched many years ago [26,27,28]. They are promising scintillators for radiation detection because the energy resolution and detection efficiencies of Y_1.8_La_0.2_O_3_ transparent ceramics can be comparable to those of NaI (Tl), CsI, and Ce^3+^:YAG scintillators. Meanwhile, they have better chemical stability and lower cost [26]. Er^3+^:Y_1.8_La_0.2_O_3_ transparent ceramics prepared by H_2_ atmosphere sintering are an ideal gain medium for the development of solid-state 1.5 μm optical amplifiers and tunable upconversion lasers [27]. It is foreseeable that Y_1.8_La_0.2_O_3_ transparent ceramics will become the core component to be used in various devices.

The fabrication of Y_2_O_3_-based transparent ceramics through conventional sintering techniques, including vacuum sintering [29], atmosphere sintering [30], and vacuum hot-press sintering [31], is significantly constrained by several inherent limitations. These methods typically require extreme processing conditions characterized by elevated sintering temperatures, prolonged dwelling times, and restricted heating rates, resulting in compromised production efficiency. In contrast, spark plasma sintering (SPS) can rapidly increase heating rates and significantly reduce processing time by pulsed-current direct heating and plasma-assisted mechanisms. However, the carbon contamination caused by the SPS equipment and graphite molds will substantially deteriorate the optical transmittance of the transparent ceramics. This fundamental constraint has consequently limited the application of SPS in the fabrication of Y_1.8_La_0.2_O_3_ transparent ceramics, as evidenced by the scarcity of relevant research publications in this field.

In this paper, Y_1.8_La_0_._2_O_3_ nanopowders as raw materials with sizes in the range of 30–60 nm were prepared by chemical co-precipitation method based on the study of Luo et al. [32]. The effects of the sintering process parameter, LiF addition, and annealing time on microstructure evolution and transmittance of SPS-sintered Y_1.8_La_0.2_O_3_ transparent ceramics were investigated.

## 2. Experimental

### 2.1. Sample Preparation

High purity Y_2_O_3_ (99.99%) and La_2_O_3_ (99.99%) powders (Shanghai Yuelong New Materials Co., Ltd., Shanghai, China), and analytically pure LiF powder (Shanghai Aladdin Science and Technology Co., Ltd., Shanghai, China) were used as raw materials for the experiments. Firstly, the Y_1_._8_La_0_._2_O_3_ precursor powders were prepared using a chemical co-precipitation method. Y_2_O_3_ and La_2_O_3_ powders were first dissolved in HNO_3_ to form a nitrate solution, and the precipitant was a mixed solution of NH_4_HCO_3_ and NH_4_OH with a molar ratio of 1:1. Using reverse titration, the titration rate was less than 3 mL/min. Then, Y_1.8_La_0.2_O_3_ powders were obtained by calcining the precursor at 900 °C for 2 h. Finally, the mixed powders Y_1.8_La_0.2_O_3_ and different amounts (0, 0.3, 0.6 wt.%) of LiF were stirred with anhydrous ethanol for 6 h and dried at 70 °C to obtain the desired powders for sintering.

**Figure 1:**
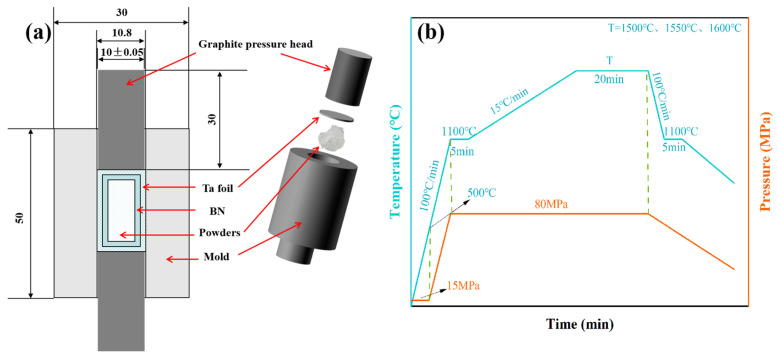
(**a**) Graphite mold cross-section and 3D drawing; (**b**) SPS process curve.

In order to prevent the reaction between powders and graphite mold, tantalum foil (thickness of 0.025 mm) sprayed with BN on the inner surface was used to separate the powders from the graphite mold (10 mm diameter), as shown in Figure 1a. The mold was placed into the SPS furnace chamber (SPS 320MK, Sumitomo, Japan), and the powders were sintered using the process shown in Figure 1b. Then, the sintered samples were obtained with a thickness of about 2 mm. Y_1.8_La_0.2_O_3_ transparent ceramics were obtained by grinding the sample to about 1 mm and were subjected to manual surface polishing using a SiO_2_ polishing suspension with a particle size of 0.06 μm, which was applied through an electric polishing machine. Finally, the transparent ceramics were annealed in an air atmosphere at 900 °C for different times (0 h (as a reference), 2 h, 3 h, and 4 h, respectively) in order to decrease the detrimental effects of carbon contamination and oxygen vacancies on the optical transmittance.

### 2.2. Characterization

The phases of Y_1.8_La_0.2_O_3_ ceramics were carried out by X-ray diffraction (D8 Advance, Bruker, Germany) with a scan rate of 2°/min between 10 and 80 (2θ angle), using Cu Kα (λ = 1.5418 Å) radiation (36 kV, 20 mA). The density of ceramics was measured by the Archimedes principle (aqueous medium). The on-line transmittance was measured by the UV-visible spectrophotometer (UV-9000s, Shanghai Yuanxi Technology Co., Ltd., Shanghai, China) in the ultraviolet range. After etching with 30 wt.% HCl for 10 min, the SE images of the ceramics were characterized by a Nova Nano SEM 450 field emission scanning electron microscope (FE-SEM, FEI, USA) operated at an acceleration voltage of 20 kV. The valence change in the O1s was analyzed using X-ray photoelectron spectroscopy (XPS, Shimadzu Kratos Axis Ultra DLD, Oxford Company, USA). The C1s binding energy was fixed at 284.6 eV of adsorbed adventitious carbons.

## 3. Results and Discussion

**Figure 2:**
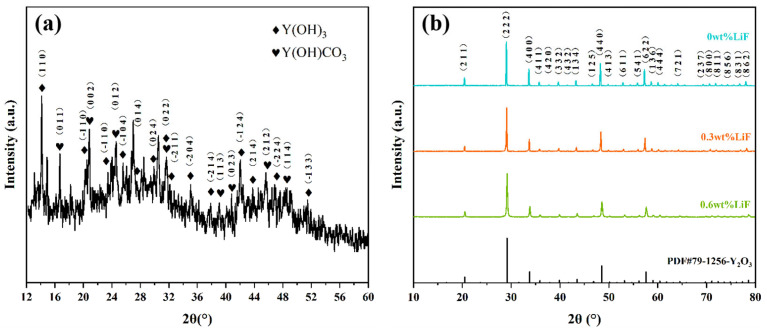
XRD patterns of Y_1.8_La_0.2_O_3_ (**a**) precursor and (**b**) transparent ceramics sintered at 1550 °C with different amounts of LiF.

Figure 2 shows the X-ray diffraction patterns of precursor and Y_1.8_La_0.2_O_3_ transparent ceramics sintered at 1550 °C doped with 0 wt.%, 0.3 wt.% and 0.6 wt.% LiF. It can be seen from Figure 2a that the precursor is mainly composed of Y (OH)_3_ (JCPDS NO. 24-1447) and Y (OH)CO_3_ (JCPDS NO. 30-1444). The unmarked peaks may be compounds of C, H, O, etc., which can be removed after calcination. As evidenced by Figure 2b, the XRD pattern of Y_1.8_La_0.2_O_3_ transparent ceramics exhibits diffraction peaks of the cubic Y_2_O_3_ phase (JCPDS NO. 79-1256) without a second phase.

**Figure 3:**
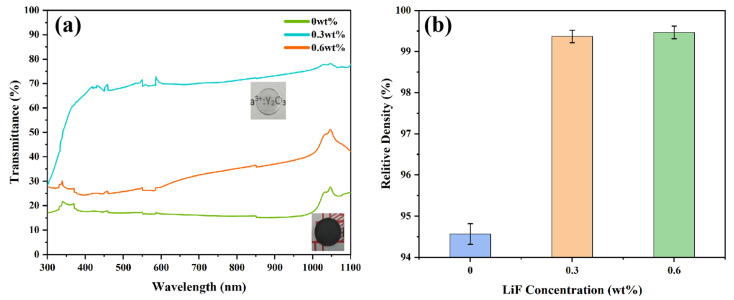
(**a**) Transmittance curves and (**b**) relative density of the Y_1_._8_La_0_._2_O_3_ ceramics sintered at 1550 °C for 20 min with different amounts of LiF.

Transmittance curves of Y_1.8_La_0.2_O_3_ ceramics at 300–1100 nm wavelength are shown in Figure 3a. The Y_1.8_La_0.2_O_3_ ceramic, without adding LiF, is black and completely opaque. Its maximum transmittance is only 23.63% due to being severely contaminated by carbon. After adding 0.3 wt.% LiF, the black part on the Y_1.8_La_0.2_O_3_ ceramic is almost invisible. The maximum transmittance is 78.10%, and the words under the ceramic can be clearly seen. This is mainly attributed to the two roles played by LiF in the SPS process. One role is the cleaning effect, which can reduce the diffusion of carbonaceous gases into ceramics [33,34] and reduce the carbon pollution of transparent ceramics. The other role is the liquid phase sintering aid. Combined with the study of Naum Frage et al. [35], the reaction process can be expressed as Equations (1) and (2), respectively:

(1) $$nC+nLiF\to {nLi}_{Y(La)}^{\prime \prime }+{nV}_{O}^{**}+{\left(CF\right)}_{n}↑$$

(2) $$2LiF\to 2{Li}_{Y(La)}^{\prime \prime }+{2F}_{O}^{*}+V_{O}^{**}$$

During sintering, LiF reacts with the carbon-based gas in the transparent ceramics to produce the volatile gas (CF)_n,_ which is shown in Equation (1). As shown in Equation (2), Li^3+^ replaces Y^3+^ and/or La^3+^, and F^−^ replaces O^2−^. Oxygen vacancies are created in ceramics to bring the electric charge to an equilibrium state according to the law of conservation of electric charge. Excess addition results in the residue of some LiF in the matrix because the absorption and scattering of light by Y_1.8_La_0.2_O_3_ ceramics lead to a decrease in transmittance due to an increase in the number of point defects [35]. The transmittance of Y_1.8_La_0.2_O_3_ ceramics decreases rapidly when 0.6 wt.% LiF is added. This may be due to the large increase in oxygen vacancies. The difference is that the large increase of oxygen vacancies reduces the transmittance of the ceramics but promotes the diffusion rate of oxygen. Oxygen is the slowest diffusion substance in Y_1.8_La_0.2_O_3_ ceramics during sintering [33]. According to the mechanism of vacancy diffusion, the large formation of oxygen vacancies favors the diffusion of oxygen in ceramics. It accelerates the densification process so that the ceramics can obtain high densities at lower sintering temperatures.

Figure 3b shows the effect of LiF contents on the relative density of Y_1.8_La_0.2_O_3_ transparent ceramics. It can be noticed that the addition of LiF increases the relative density of the ceramics. The relative density of Y_1.8_La_0.2_O_3_ ceramics dropped by 0.3 wt.% and 0.6 wt.% LiF are all above 99%. This is attributed to the effect of LiF liquid phase sintering, which increases the possibility of grain re-arranging [36].

**Figure 4:**
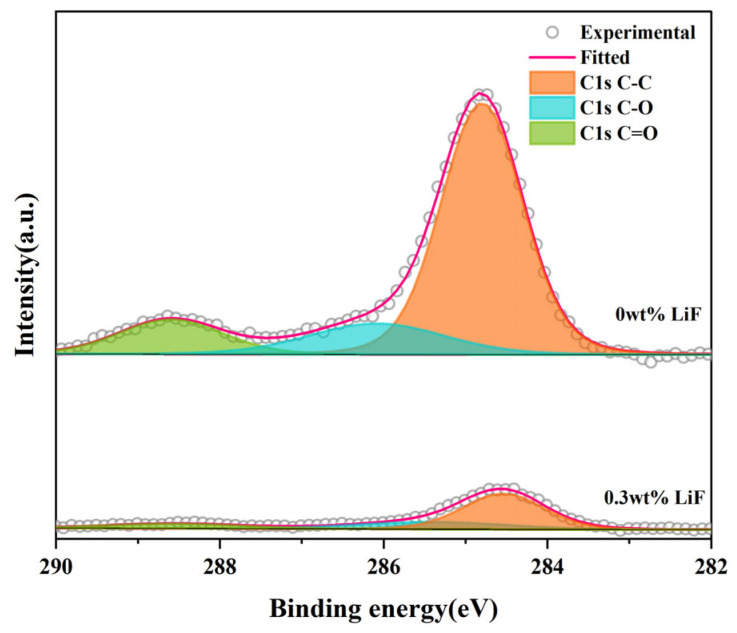
The C1s spectra of theY_1_._8_La_0_._2_O_3_ transparent ceramics doped with 0.3wt%LiF and undoped LiF before annealing at 900 °C for 3h.

Figure 4 shows the C1s spectra of the Y_1.8_La_0.2_O_3_ transparent ceramics doped with 0.3 wt.% LiF and undoped LiF before annealing at 900 °C for 3 h. After comparing the intensity of the C1s peaks of the two transparent ceramics, it can be found that the carbon content of Y_1.8_La_0.2_O_3_ transparent ceramics decreased significantly [37] after doping 0.3 wt.% LiF, which indicates that the doping of LiF can reduce carbon pollution during the SPS process.

**Figure 5:**
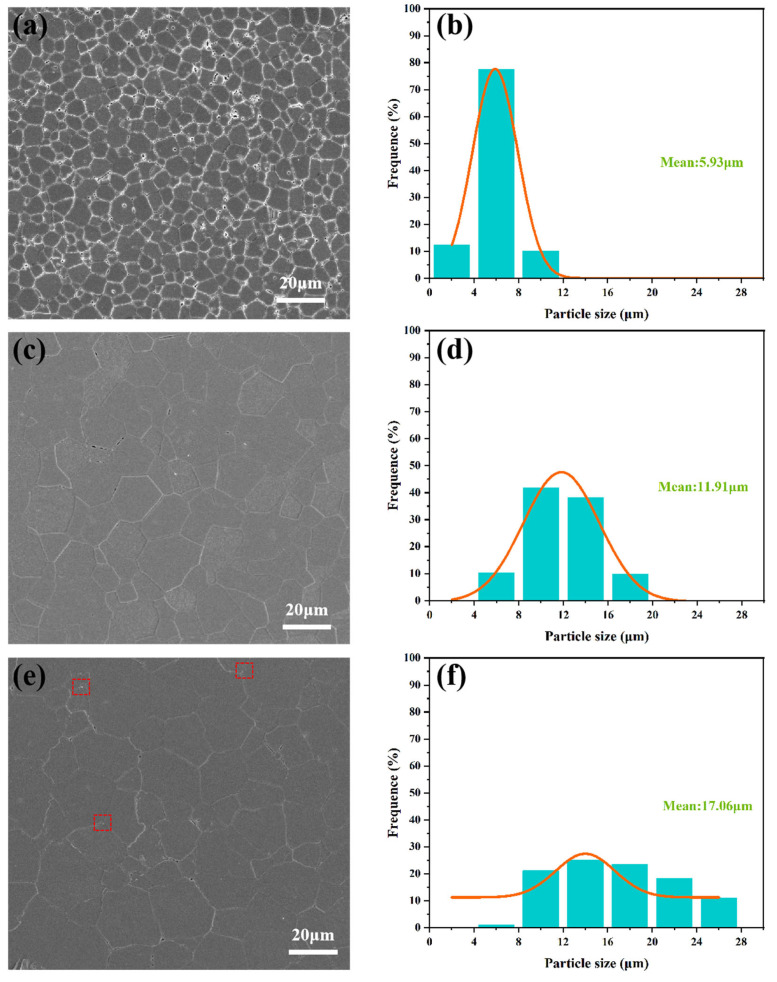
Microstructure and grain size distribution of Y_1.8_La_0.2_O_3_ ceramics sintered at 1550 °C with (**a**,**b**) 0 wt.% LiF, (**c**,**d**) 0.3 wt.% LiF, and (**e**,**f**) 0.6 wt.% LiF.

It is well known that porosity is a key factor in the transmittance of ceramics. Figure 5a shows the microstructure of Y_1.8_La_0.2_O_3_ transparent ceramics without adding LiF. It can be clearly seen that there are many holes distributed at the grain boundaries and inside the grains. The phenomenon can be explained by the low transmittance of the ceramics by Mie scattering [38]. Its scattering coefficient can be expressed by Equation (3):

(3) $$S_{m}=\frac{CNV}{\frac{\lambda -k\alpha_{0}^{2}}{\alpha_{0}}}$$

In the formula, C and k are constants, α_0_ is the size of the scattering center, V is the volume of the stomata, and N is the number of scattering centers. It can be found that the scattering coefficient of ceramics is proportional to the grain size, volume, and number of air holes. For the Y_1.8_La_0.2_O_3_ transparent ceramic without adding LiF, the high number and size of pores have a high optical scattering coefficient, causing it to exhibit an opaque state. Figure 5c and e show the microstructure of Y_1.8_La_0.2_O_3_ transparent ceramics doped with 0.3 and 0.6 wt.% LiF, respectively. Almost no pores can be observed at grain boundaries and inside the grains, which is consistent with the previously analyzed phenomenon that oxygen vacancies can promote the densification of ceramics. The reduction in the number of pores leads to a decrease in the optical scattering coefficient and an increase in the transmittance of the ceramics. The grain size bars obtained by the measurements of about 200 grain sizes for each group are shown in Figure 5b, 5d and 5e, respectively. It can be seen that the mean grain size increases obviously with the increase in LiF addition.

**Figure 6:**
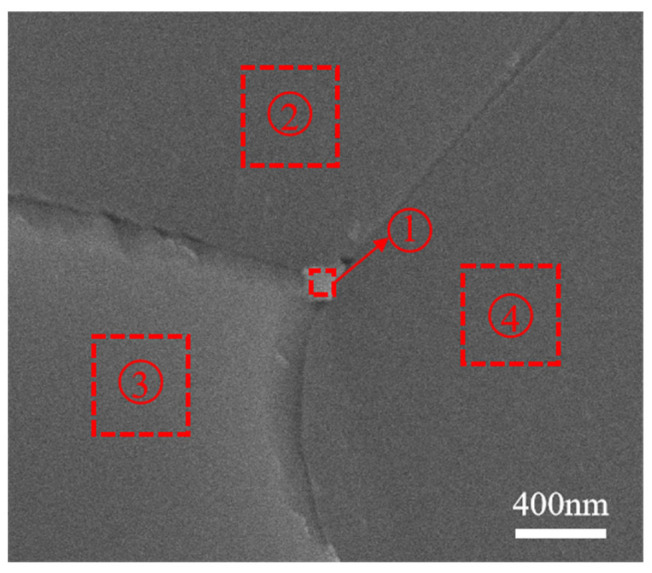
Microstructure of Y_1.8_La_0.2_O_3_ transparent ceramics with 0.6 wt.% LiF.

It is worth noting that some small white particles can be observed at the grain boundary in Figure 5e (framed part of the figure). This phenomenon is not found in Figure 5a,c. After continuing to zoom in at the framed upper right corner of Figure 5e, a white particle can be found at the grain boundary, as shown in Figure 6. The results of the composition of the matrix particles and white particles tested are listed in Table 1. The amount of F is significantly higher in region 1 than in regions 2, 3, and 4. It indicates that most of the F^−^ reacts with C and volatilized to produce (CF)_n_ gas, while an excess of F^−^ is preferentially present in the small white particles at the grain boundaries rather than in the matrix. This is consistent with the research result of Marder R [39]. In reference to some related studies [35,36,40], it is believed that this element is related to another light element (like Li) that cannot be detected by EDS due to the impossibility of F^−^ to exist alone. Therefore, it is reasonable to believe that after adding excessive sintering aids, part of LiF will segregate at the grain boundary to form a second phase, which enhances the light absorption and scattering of ceramics and reduces the transmittance of transparent ceramics. The other part of LiF generates volatile gas (CF)n or enters into the crystal lattice as described in Equations (1) and (2).

**Figure 7:**
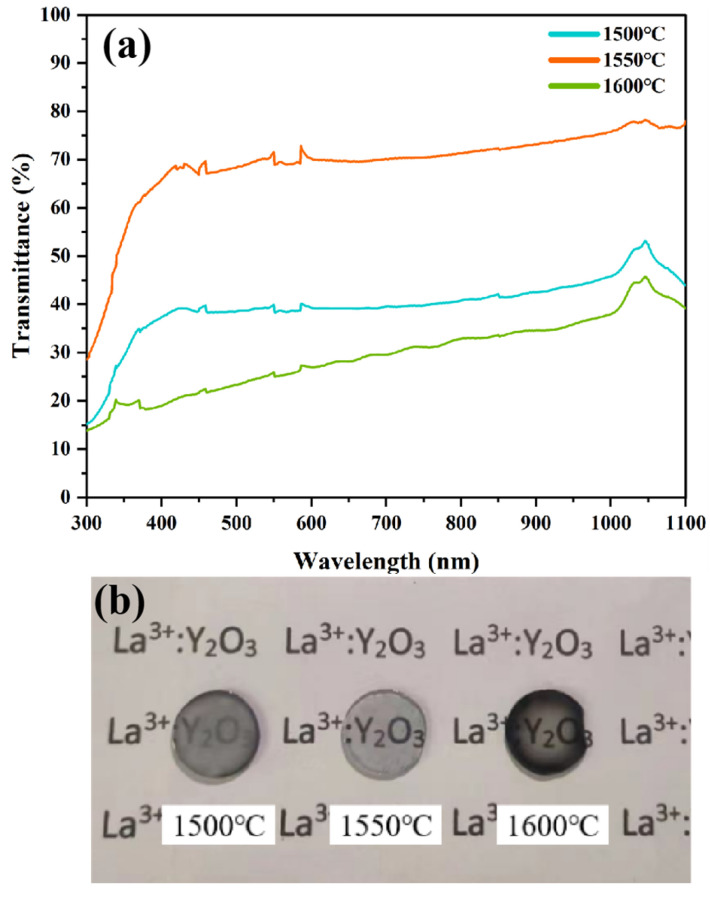
(**a**) Transmittance curve and (**b**) physical picture of Y_1.8_La_0.2_O_3_ ceramics sintered at different temperatures with 0.3 wt.% LiF.

Figure 7a shows the transmittance curves of Y_1.8_La_0.2_O_3_ ceramics doped with 0.3 wt.% LiF sintered at different sintering temperatures. The transmittance increases when the sintering temperature increases from 1500 °C to 1550 °C. It is mainly due to the decrease in the total grain boundary area through the gradual growth of the grains, which can decrease the light scattering loss and the optical scattering coefficient of the ceramics [41]. The transmittance of the ceramic, whose central part is dark yellow and the surrounding part is black (Figure 7b), decreases rapidly when the sintering temperature increases to 1600 °C. The reason for this phenomenon may be that the higher sintering temperature leads to the enhancement of carbon diffusion, which makes the ceramics suffer more serious carbon pollution.

**Figure 8:**
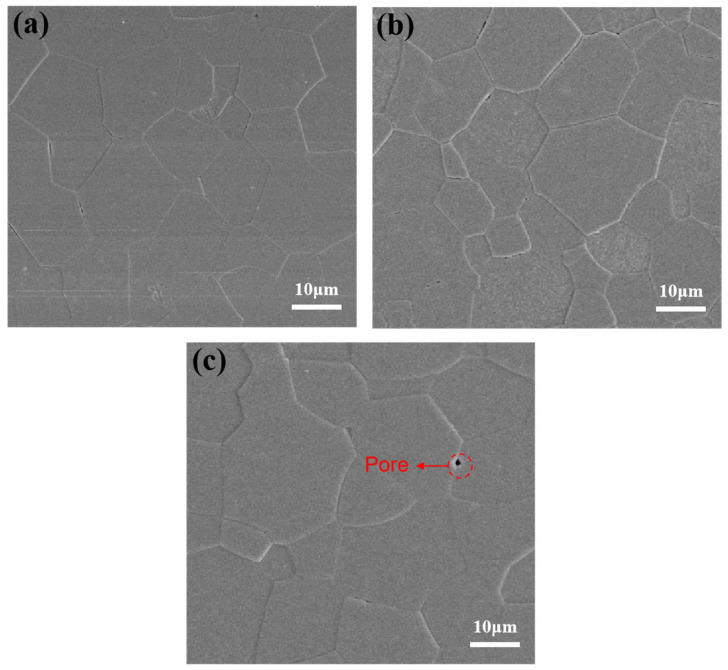
Microstructure of Y_1.8_La_0.2_O_3_ ceramics with 0.3 wt.% LiF sintered at (**a**) 1500 °C, (**b**) 1550 °C and (**c**) 1600 °C, respectively.

In general, the densification process of SPS can be divided into three stages [42]. The first stage is described by the accumulation of grains. The second stage is related to the diffusion process that accompanies neck formation and grain sliding. The third stage is the elimination of pores, mainly through diffusion at grain boundaries. Figure 8 shows the microstructure of Y_1.8_La_0.2_O_3_ ceramics prepared at different sintering temperatures for 20 min. It can be seen that the microstructure of the ceramic sintered at 1500 °C is dense, and no obvious pores exist. The particle size is about 10–25 μm. The high densities of transparent ceramics are due to two reasons: one is the liquid phase sintering effect of LiF, and the other is related to the sintering method. In addition to the Joule heat generated by energization and the plastic deformation generated by pressurization, the SPS method can effectively reduce the densification temperature by effectively utilizing the self-heating effect generated by the powder particle-to-particle discharge. That is to say, the densification of the ceramics has entered the third stage at 1500 °C. The grain size grows slightly when the sintering temperature increases to 1550 °C. At the same time, some larger pores can be observed when the sintering temperature is 1600 °C. It may be due to the fact that the higher sintering temperature gives enough driving force to the grain boundaries to move faster during the grain growth process, resulting in a small portion of the gas being retained inside the ceramic during the sintering process. On the whole, the grain size of the ceramics gradually increases when the sintering temperature increases from 1500 °C to 1600 °C. However, the grain size changes slightly and is in the range of 10–30 μm.

**Figure 9:**
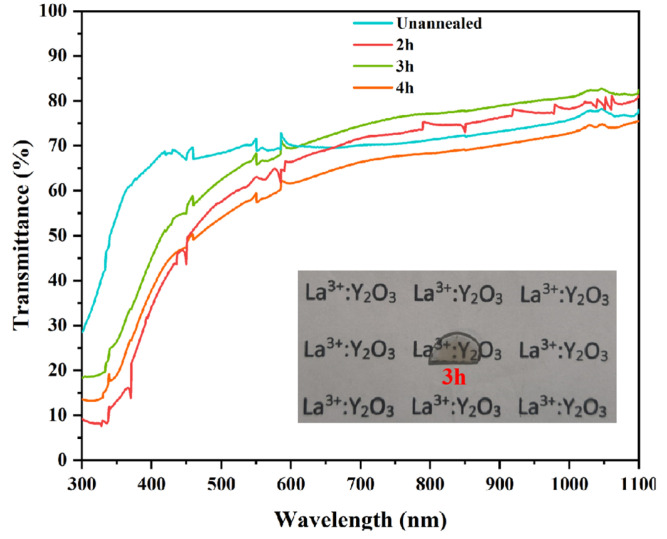
Transmittance curves of Y_1.8_La_0.2_O_3_ ceramics with 0.3 wt.% LiF annealed at 900 °C for different time.

Although oxygen vacancies can promote the densification of ceramics, they also cause a decrease in the transmittance of ceramics [43]. Annealing can reduce the number of oxygen vacancies and the residual carbon pollution in transparent ceramics because the partial pressure of oxygen in the air is higher than inside the transparent ceramics, and the high temperature provides enough diffusion power to make the combination of oxygen atoms and oxygen vacancies [44]. Figure 9 shows the transmittance curves of Y_1.8_La_0.2_O_3_ transparent ceramics with 0.3 wt.% LiF is annealed at 900 °C for different time. Compared to the unannealed samples, the transmittance increases after annealing for 2 h and 3 h, respectively. The best transmittance of 82.67% can be obtained by annealing for 3 h. The transmittance decreases slightly after annealing for 4 h.

**Figure 10:**
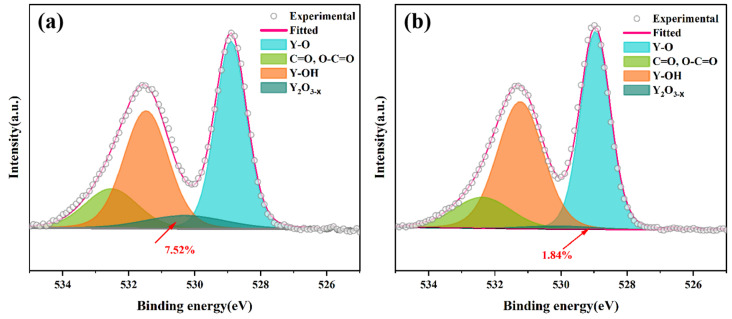
The O1s spectra of theY_1.8_La_0.2_O_3_ transparent ceramics with 0.3 wt.% LiF (**a**) before and (**b**) after annealing at 900 °C for 3 h.

Figure 10 shows the O1s spectra of the sintered Y_1_._8_La_0_._2_O_3_ transparent ceramics and annealed Y_1.8_La_0.2_O_3_ transparent ceramics at 900 °C for 3 h, respectively. A subpeak of reduced yttrium oxide is found in the region around 530.4 eV [45]. It can be found that the area ratio occupied by the subpeak of reduced yttrium oxide decreases from 7.52% to 1.84% after annealing at 900 °C for 3 h. This indicates that annealing can effectively remove the oxygen vacancies inside the transparent ceramics.

## 4. Conclusions

Y_1.8_La_0.2_O_3_ transparent ceramics were prepared by SPS using commercial Y_2_O_3_, La_2_O_3,_ and LiF as raw material at 1500~1600 °C under the pressure of 80 MPa for 20 min. LiF acts as a carbon removal by reacting with carbon to form (CF)_n_ gas and a sintering aid by liquid phase sintering during the SPS process. Excess addition of LiF will form a second phase at the grain boundaries and reduce the transmittance of the ceramics. The relative density and transmittance of Y_1.8_La_0.2_O_3_ ceramics doped by 0.3 wt.% LiF sintered at 1550 °C are 99.36% and 78.10%, respectively. When the sintering temperature reaches 1600 °C, the transmittance of the ceramics decreases due to the serious carbon contamination. Proper annealing can increase the transmittance by reducing the oxygen vacancy content. The Y_1.8_La_0.2_O_3_ transparent ceramics can obtain the maximum transmittance of 82.67% in the UV wavelength after annealed at 900 °C for 3 h.

## Figures and Tables

**Table 1:** Micro-area EDS analysis of Y_1.8_La_0.2_O_3_ ceramics sintered at 1550 °C for 20 min with 0.6 wt.% LiF.

Elements	Point 1 (at%)	Point 2 (at%)	Point 3 (at%)	Point 4 (at%)
Y	34.24	36.37	35.09	34.38
La	3.30	3.60	3.31	3.72
F	3.62	0	0.27	0.23
O	58.83	60.03	61.33	61.67

## Data Availability

The original contributions presented in this study are included in the article. Further inquiries can be directed to the corresponding author.
